# Exploring Serum Zinc and Copper Levels as Potential Biomarkers in Polycystic Ovary Syndrome: A Cross-Sectional Study From Northeast India

**DOI:** 10.7759/cureus.57393

**Published:** 2024-04-01

**Authors:** Jayanta Das, Bidyut Bhuyan, Pawan Kumar, Chandan Nath, Himangshu Malakar, Purnima Rajkhowa, Polina Boruah

**Affiliations:** 1 Biochemistry, All India Institute of Medical Sciences, Guwahati, Guwahati, IND; 2 Biochemistry, Tezpur Medical College, Tezpur, IND; 3 Obstetrics and Gynaecology, All India Institute of Medical Sciences, Guwahati, Guwahati, IND; 4 Microbiology, All India Institute of Medical Sciences, Guwahati, Guwahati, IND; 5 Biochemistry, North Eastern Indira Gandhi Regional Institute of Health and Medical Sciences, Shillong, IND

**Keywords:** association, biomarker, zinc, copper, polycystic ovary syndrome (pcos)

## Abstract

Introduction

Polycystic ovary syndrome (PCOS) is a prevalent hormonal disorder characterized by irregular menstrual cycles, ovarian cysts, and elevated androgen levels. The potential association between trace elements, specifically copper (Cu) and zinc (Zn), and PCOS has been explored, but a definitive relationship remains unclear. This study aims to investigate the levels of these trace elements in women with PCOS and their potential implications.

Methods

The study, conducted at Gauhati Medical College & Hospital, involved 60 individuals with PCOS and a matched control group. Ethical approval was obtained, and participants provided written informed consent. The study spanned from July 2021 to June 2022, utilizing a hospital-based case-control study design. Diagnostic criteria adhered to the Rotterdam criteria, and serum copper and zinc levels were quantified using a double-beam UV spectrophotometer.

Results

In the PCOS group, the mean age was 23.01 ± 3.60 years, while the control group had a mean age of 23.34 ± 3.59 years, with no significant age difference. Mean copper levels were 147.32 ± 16.53 μg/dl in PCOS and 106.88 ± 15.60 μg/dl in controls, indicating a significant increase in PCOS (p < 0.0001). Mean zinc levels were 93.99 ± 6.76 μg/dl in PCOS and 85.42 ± 12.69 μg/dl in controls, also significantly higher in PCOS (p < 0.0001).

Conclusion

The study highlights significant differences in serum copper and zinc levels between women with PCOS and healthy controls, suggesting potential implications for the syndrome's pathophysiology. Further research is warranted to elucidate the precise roles of these trace elements in PCOS and explore therapeutic interventions.

## Introduction

Polycystic ovary syndrome (PCOS) is a prevalent hormonal disorder affecting women and is characterized by irregular menstrual cycles, ovarian cysts, and elevated androgen levels [1,2]. First described by Stein and Leventhal in 1935, PCOS has evolved into one of the most prevalent endocrine disorders encompassing hyperandrogenism, menstrual irregularities, infertility, and hirsutism [3]. While the direct relationship between trace elements, specifically copper (Cu) and zinc (Zn), and PCOS remains unclear, ongoing research has explored their potential roles in the context of this complex syndrome [4,5].

Zinc, known for its involvement in androgen and insulin metabolism, has been the focus of studies investigating its levels in women with PCOS. Some findings suggest a potential association between lower zinc levels and PCOS [6], prompting exploration into zinc supplementation as a therapeutic avenue for addressing insulin sensitivity [7] and androgen reduction [8] in affected individuals.

On the other hand, copper, an essential component in enzymatic reactions and protein structure, has yet to demonstrate a direct link to PCOS [9]. However, the intricate interplay between copper and zinc, with their often-interdependent relationship, raises questions about their combined impact on overall health and its potential relevance to PCOS.

It is crucial to acknowledge that research in this area is dynamic, and a definitive relationship between copper, zinc, and PCOS is yet to be established. Individual responses to trace elements can vary, influenced by factors such as diet, genetics, and lifestyle. As we delve into the complex interconnections between zinc levels and PCOS, exploring potential factors like insulin resistance, androgen levels, inflammation, dietary habits, and oxidative stress becomes integral. For those concerned about zinc levels in the context of PCOS, seeking guidance from healthcare professionals, who can conduct specific tests and provide personalized advice, is recommended. Additionally, addressing broader nutritional and lifestyle factors remains pivotal in the comprehensive management of PCOS and overall health.

## Materials and methods

The collaborative investigation conducted by the Department of Biochemistry and the Department of Obstetrics & Gynecology at Gauhati Medical College & Hospital in Guwahati received ethical approval from the Institutional Ethical Committee. Written informed consent, ensuring participants' comprehension in their native language, was obtained for the study, which spanned from July 2021 to June 2022. The research, employing a hospital-based case-control study design, focused on individuals aged 18 to 40 years seeking health care at the Obstetrics and Gynecology Department, diagnosed with PCOS. The diagnostic criteria followed the Rotterdam criteria, necessitating women to meet at least two out of three criteria: oligomenorrhea and/or amenorrhea, clinical and/or biochemical signs of hyperandrogenism, and polycystic ovaries observed on ultrasonography [10].

The quantification of serum copper and zinc levels utilized a double-beam UV spectrophotometer (spectra scan UV2600), with reagent kits supplied by Tulip Diagnostics (P) Ltd (Kalyanpur, India), intended for in vitro diagnostics. Calibration was meticulously performed using calibrators from the manufacturer, ensuring accuracy. Quality control measures were strictly adhered to, employing a two-level Bio-Rad internal quality control system (Hercules, CA). The normal reference values for copper are 80-140 μg/dl for adult males, 80-155 μg/dl for adult females, and 30-150 μg/dl for children up to 10 years. Meanwhile, the normal range for adult serum zinc concentration is 60-120 μg/dl.

The data were analyzed using SPSS version 22.0 software (IBM Corp., Armonk, NY). Continuous data were presented as mean (with standard deviation (SD)) or median (with range). In all calculations, a two-tailed p-value < 0.0001 was regarded as statistically significant.

## Results

In the present study, we conducted a comprehensive study involving 60 individuals diagnosed with PCOS through clinical (Rotterdam criteria 2003) and ultrasonographic assessments. The subjects were consecutively selected from patients attending Gauhati Medical College & Hospital during the period from July 2021 to June 2022. These cases were meticulously examined and systematically compared with a control group consisting of 60 individuals matched for age and sex over the same timeframe.

Within the case group, comprising 60 individuals, the mean age and standard deviation of the subjects were calculated to be 23.01 ± 3.60 years, with a median age of 22 years. Conversely, the control group, also comprising 60 individuals, exhibited a mean age and standard deviation of 23.34 ± 3.59 years, with a median age of 23 years (Table 1). Importantly, statistical analysis revealed no significant difference in age between the case and control groups, ensuring a well-matched comparison.

**Table 1:** Frequency of age distribution of the study group

Age distribution (years)	Case (n = 60)	Control (n = 60)	Mean age (case) = 23.01 ± 3.60
15-20	18 (30%)	19 (31.7%)	
21-25	25 (41.7%)	23 (38.3%)	
26-30	15 (25%)	26 (43.3%)	Mean age (control) = 23.34 ± 3.59
31-35	1 (1.7%)	1 (1.7%)	
36-40	1 (1.7%)	1 (1.7%)	

**Figure 1:**
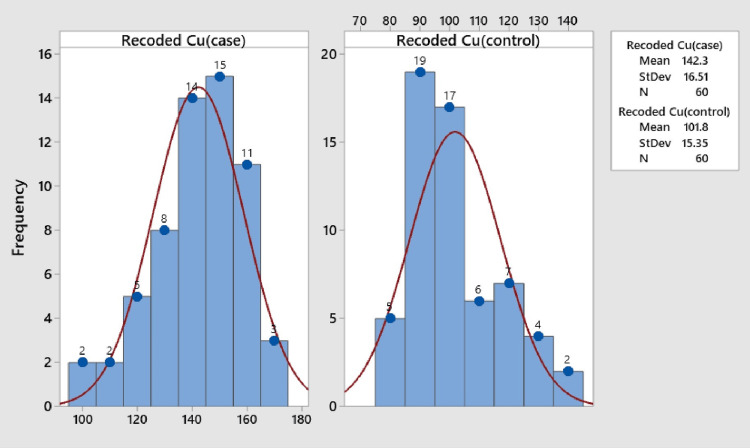
Mean copper levels in case and control groups: insights from histogram analysis

Exploring the biochemical parameters, the mean copper levels in the case and control groups were determined as 147.32 ± 16.53 μg/dl and 106.88 ± 15.60 μg/dl, respectively. The data highlighted a distinct pattern, with the maximum copper level observed in the case group falling within the range of 145-155 μg/dl, whereas the control group exhibited a maximum copper level between 85 and 95 μg/dl (Figure 1).

Significantly, the mean copper level in the case group displayed a substantial increase compared to the control group, supported by a p-value < 0.0001, signifying statistical significance (Table 2).

**Table 2:** Serum level of copper and zinc in the study population

Parameter	Case (n = 60)		Control (n = 60)		P-value
	Mean	SD	Mean	SD	
Serum copper (μg/dl)	147.32	16.53	106.88	15.60	<0.0001
Serum zinc (μg/dl)	93.99	6.76	85.42	12.69	<0.0001

**Figure 2:**
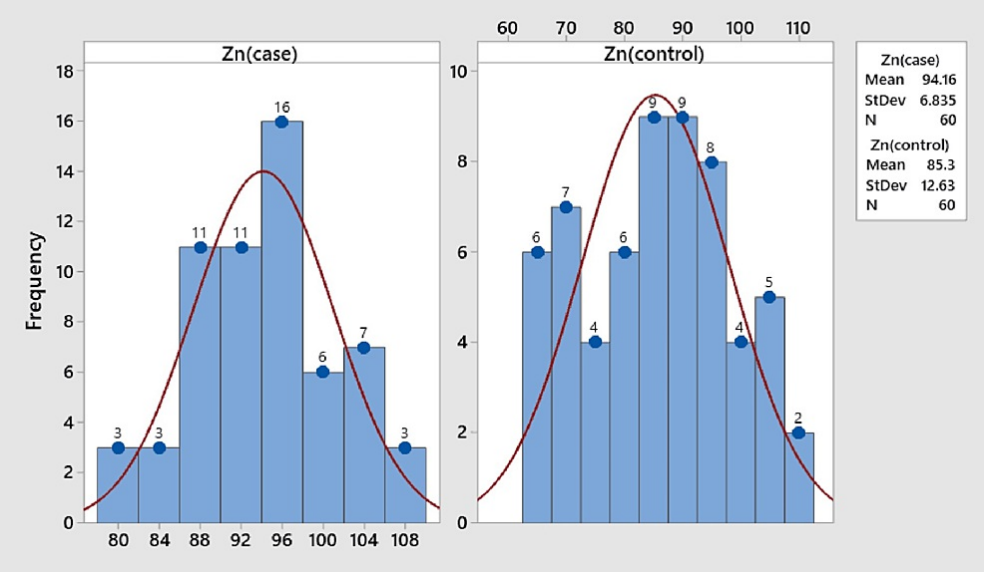
Mean zinc levels in case and control groups: insights from histogram analysis

Turning attention to zinc levels, the mean zinc concentrations in the case and control groups were reported as 93.99 ± 6.76 μg/dl and 85.42 ± 12.69 μg/dl, respectively. The maximum zinc level in the case group was identified within the range of 94-98 μg/dl, while the control group's maximum zinc level ranged between 82.5 and 92.5 μg/dl (Figure 2).

Remarkably, the mean zinc level in the case group exhibited a significant increase compared to the control group, supported by a p-value < 0.0001 (Table 2).

## Discussion

In recent years, heightened attention has been directed toward understanding the intricate relationship between micro and macro elements and their impact on human health. A study by Benaglia et al. has underscored the connection between abnormal element metabolism and various gynecological conditions, including infertility, recurrent abortion, and malignancy [9]. Surprisingly, mineral levels in women with PCOS have not undergone comprehensive analysis. Among these essential micronutrients, zinc and copper play pivotal roles in the function of metalloenzymes and proteins governing cellular metabolism and pathways regulating oxidative stress. Our hypothesis posits a potential link between these trace elements and PCOS, specifically in the context of oxidative stress.

Zinc, a micronutrient integral to glucose metabolism, insulin synthesis, secretion, and signaling, emerges as a focal point in the development of PCOS and its metabolic complications. Previous studies have suggested lower zinc levels in women with PCOS compared to their healthy counterparts [10], with no difference [11] or higher zinc levels [12]. However, the exact cause, whether stemming from inadequate intake, absorption issues, heightened excretion, or increased zinc requirements, remains unclear. Zinc deficiency has been associated with diminished antioxidant capacity, leading to insulin resistance and apoptosis [13]. Notably, our study reveals a significantly higher serum zinc content (93.99 ± 6.76 μg/dl) in women with PCOS compared to healthy individuals. The potential implication is that higher zinc levels may destabilize insulin hexamers and hinder their storage in the pancreas, particularly in women with insulin resistance.

Copper, an essential element for red blood cell formation, bone health, and connective tissue, has also captured attention in the context of PCOS [14]. A systematic review and meta-analysis conducted by Yin et al. revealed a notable elevation in copper levels among women with PCOS compared to healthy controls. The standardized mean difference (SMD) was determined to be 0.52, indicating a statistically significant difference, with a 95% confidence interval of 0.38 to 0.67. The analysis demonstrated a moderate level of heterogeneity, as indicated by an I2 value of 47.6% [15]. In another systematic review and meta-analysis by Jiang et al., encompassing nine studies with a total of 1,168 PCOS patients and 1,106 controls, the pooled effect size indicated a significant elevation in serum copper levels among women with PCOS (SMD = 0.51 μg/mL, 95% CI = 0.30, 0.72; p < 0.0001) [16]. Literature suggests a link between heightened copper levels and PCOS, with copper ions acting as catalysts in the synthesis of reactive oxygen species (ROS), thereby inducing oxidative stress [17,18]. Prolonged exposure to copper has been associated with ovarian dysfunction. Additionally, copper combined with homocysteine increases the risk of early vascular diseases in women with PCOS [18]. Our study found serum copper levels ranging from 147.32 ± 16.53 (μg/dl), deviating from the reference range for adult males (80-140 μg/dl). Interestingly, unlike studies associating elevated serum copper with obesity, our findings did not establish such a correlation in women with PCOS.

Limitations

Our study has a few limitations that may impact the interpretation and generalizability of the findings. Firstly, the study's cross-sectional design restricts the establishment of causal relationships, as it captures data at a single point in time. Longitudinal studies could provide a more comprehensive understanding of the dynamic interactions between trace elements and PCOS over time. Secondly, the relatively small sample size of 60 individuals with PCOS and matched controls may limit the study's statistical power and affect the robustness of the observed associations. A larger and more diverse participant pool could enhance the study's external validity and strengthen the reliability of the results. Additionally, our study focuses on serum copper and zinc levels, while informative, does not encompass a broader exploration of other potential contributing factors to PCOS. Future investigations incorporating a more comprehensive array of variables, such as dietary habits, genetic predispositions, and lifestyle factors, would provide a more holistic understanding of the syndrome's etiology.

## Conclusions

In summary, our exploration into the intricate dynamics of zinc and copper levels in women with PCOS reveals significant deviations, shedding light on potential mechanisms contributing to the syndrome's pathophysiology. The nuanced interplay of these essential trace elements in the context of PCOS warrants further investigation to unravel their precise roles and implications for therapeutic interventions.
